# Saponin from *Periploca forrestii* Schltr Mitigates Oxazolone-Induced Atopic Dermatitis via Modulating Macrophage Activation

**DOI:** 10.1155/2020/4346367

**Published:** 2020-10-15

**Authors:** Luting Zeng, Yingqin Liu, Congcong Xing, Yijie Huang, Xin Sun, Guangchen Sun

**Affiliations:** ^1^Pharmacy College, Guilin Medical University, No. 109 North Second Huancheng Road, Qixing District, Guilin, 541004 Guangxi, China; ^2^Biotechnology College, Guilin Medical University, Guilin, Guangxi, China; ^3^Medical College, Xiamen University, Xiamen, Fujian, China

## Abstract

Atopic dermatitis (AD) is a relapsing, acute, and chronic skin disease featured by intractable itching, eczematous skin. Conventional therapies based on immunosuppression such as corticosteroids are associated with multiple adverse reactions. *Periploca forrestii* Schltr saponin (PFS) was shown to potently inhibit murine arthritis by protecting bone and cartilage injury and suppressing NF-*κ*B activation. However, its therapeutic effect on oxazolone-induced atopic dermatitis (AD) and the underlying mechanisms on macrophage are still unclear. The AD-like dermatitis was induced by repeated oxazolone challenge to the skin of BALB/c mice in vivo. Blood and ears were biochemically or histologically processed. RT-PCR, western blotting, and ELISA were conducted to evaluate the expression of macrophage factors. Mouse bone marrow-derived macrophages (BMDMs) stimulated with lipopolysaccharide (LPS) were used as a model in vitro. PFS treatment inhibited AD-like dermatitis development. PFS downregulated epidermis thickness and cell infiltration, with histological analysis of the skin lesion. PFS alleviated plasma immunoglobulin (Ig) E, IgG2a, and IgG1 levels. PFS downregulated the expression of M1 macrophage factors, tumor necrosis factor- (TNF-) *α*, interleukin- (IL-) 6, monocyte chemotactic protein-1 (MCP-1), and nitric oxide synthase2 (NOS2), and M2 macrophage factors, IL-4, arginase1 (Arg1) and CD163 in AD-like skin, which were confirmed by western blot and ELISA analysis. In addition, PFS inhibited LPS-induced macrophage polarization via the inhibition of the phosphorylation of signal transducer and activator of transcription 3 (STAT3) and nuclear translocation of NF-*κ*B p65. These results suggest that PFS exerted an antidermatitis effect against oxazolone by modulating macrophage activation. PFS administration might be useful in the treatment of AD and inflammatory skin diseases.

## 1. Introduction

Atopic dermatitis (AD) is a relapsing, acute, and chronic skin disease featured by intractable itching, eczematous skin, including skin structural defects and immune disorders. AD affects about 25% of children and 2-10% of adults; its prevalence is increasing worldwide [1, 2]. Many patients with severe atopic dermatitis have increased T helper (Th) cell reactivity; sensitization to allergens; elevated IgE-mediated reactivity to allergens; increased blood eosinophils; changes in the skin barrier function, in some cases associated with mutations in the filaggrin gene; and increased colonization of Staphylococcus aureus [3]. The skin barrier disruption is susceptible to environmental allergens, irritants, and microbes and triggers an inflammatory cascade [4]. Multiple inflammatory pathways are involved in the immune dysregulation of AD. The inflammatory response is mainly mediated by Th2 in the acute phase and with additional involvement of Th1, Th17, and Th22 cells in chronic disease [5–8]. Activated Th2 cells release IL-4 and IL-13, promoting the production of antigen-specific IgE in B cells, through the signal transducer and activator of transcription (STAT) pathway [9]. In addition, antigen-presenting cells such as macrophages play key roles in the development of AD. In response to microenvironments at the site of inflammation, macrophages give rise to different polarization with distinct functions that are categorized as classical M1 macrophages and alternative M2 macrophages. M1 macrophages, induced by lipopolysaccharides (LPS) and interferon- (IFN-) *γ*, are mainly involved in the expansion of inflammation and high antigen-presenting capabilities, and they produce proinflammatory cytokines (e.g., TNF-*α*, IL-6, and IL-12) and trigger Th1-polarized responses. In contrast, M2 macrophages, induced by IL-4 and IL-13, have anti-inflammatory and immunoregulatory functions and are characterized by the expression of Arg1, cluster of differentiation (CD)163, and mannose receptor (CD206) [10, 11]. However, it was reported that macrophages increase in both acute and chronically inflammatory AD skin [12]. Because of their versatile roles in the pathophysiology of AD, their capacities to promote and prevent the expression of allergic skin inflammation, macrophages may comprise a promising cell target for AD therapy in the future [13]. The exact mechanism of macrophage in chronic inflammatory disease such as AD is still not well understood.

Immunosuppressive agents (oral corticosteroids, cyclosporin A, etc.) have variable efficacy for the treatment of AD and are associated with multiple adverse reactions upon prolonged use [3, 14, 15]. The other agents such as tacrolimus and pimecrolimus, or phosphodiesterase-4 inhibitors are promising for the treatment of AD. However, case reports and animal carcinogenicity studies show that exposure to these agents may increase the possible risk of malignancy [15–19]. Therefore, it is necessary to develop a therapeutic tool capable of alleviating the clinical symptoms of AD as well as reducing side effects. Alternative medicine exerts pharmacological effects on atopic dermatitis, especially in preventing disease recurrence, maintaining long-term remission, and reducing disease burden; nowadays, both monomer preparations and traditional formulas are still widely used [20]. Periploca forrestii Schltr (P. forrestii) is the roots and rhizomes of a plant from the Asclepiadaceae family and widely utilized for traditional folk medicine to treat rheumatoid arthritis, bruises, and fractures [21]. Recent studies have indicated that the cardiac glycosides as a potential active ingredient of P. forrestii, promote wound healing [21–23]. Several in vivo and in vitro studies of P. forrestii or P. forrestii-containing Chinese medicine have also focused on delineating their effects on antiarthritis activities [24–26]. In addition, P. forrestii also exert an anti-inflammatory effect, antiallergy activities, and antioxidant activities [27–29].

Our previous studies demonstrated that *P. forrestii* saponin (PFS) inhibits murine arthritis by suppressing cytokine production, local inflammation, and systemic autoimmune responses; PFS and its active compound periplocin also inhibit osteoclastogenesis by reducing proteinases and phosphorylation of NF-*κ*B p65 and its inhibitory protein (I*κ*B) [30, 31]. In the present study, we further affirmed the anti-AD effect of PFS using the oxazolone-induced AD-like mouse model and elucidated the underlying molecular mechanisms of its action, providing evidence of the utilization of PFS for treating AD. Moreover, the effects of PFS were investigated in LPS-induced macrophage polarization in vitro. The study on mechanisms also elucidated that macrophage activation was involved in the action of PFS for alleviating AD.

## 2. Materials and Methods

### 2.1. Reagents and Antibodies

*Periploca forrestii* Schltr were obtained from Yulin, Guangxi, China. A voucher specimen was deposited in the Medicinal Herb Garden, Guilin Medical University. Periplocin was purchased from General Administration of Quality Supervision, Inspection and Quarantine China. Oxazolone was purchased from Alfa Aesar Thermo Fisher. Hydrocortisone (HC), *α*-MEM, MTT, and phosphatase inhibitors were purchased from Beijing Solarbio. Lipopolysaccharide (LPS, Escherichia) was purchased from Sigma-Aldrich, and fetal calf serum was purchased from Equus Biotechnology. M-CSF was purchased from PeproTech, USA. Protease inhibitor cocktail was from MCE, USA. ELISA kits for IgE, IgG1, IgG2a, TNF-*α*, MCP-1, IL-6, IL-4, IL-10, and IL-22 were purchased from Thermo Fisher. Antibodies for NOS2 (sc-7271), STAT3 (sc-8019), and MCP-1 (sc-52701) were purchased from Santa Cruz Biotechnology, Arg1 antibody (CST-93668) was obtained from Cell Signaling Technology, CD163 (ab182422) antibody was purchased from Abcam, p-STAT3 Tyr705 antibody (Tyr705-11045) was purchased from Signalway Antibody, and GAPDH antibody (60004-1-Ig) was purchased from Proteintech. Horseradish peroxidase- (HRP-) conjugated rabbit, mouse, or rat IgG secondary antibodies (ZB-2301, ZB-2305, and ZB-2307) were purchased from ZSGB-BIO, China. Primers were synthesized from Invitrogen.

### 2.2. Preparation of Periploca forrestii Schltr Saponin

The dry *Periploca forrestii* Schltr roots were ground into powder. The powders were socked with 70% ethanol for 2 weeks and refluxed twice. Then, the extract was filtered and evaporated under reduced pressure. The concentrate was extracted with petroleum ether, followed by n-butanol extraction. The n-butanol extract was concentrated and lyophilized to yield PFS powder.

### 2.3. Chromatographic Conditions

The content of periplocin in PFS was analyzed using an Agilent 1290-6495 Accurate-Mass Q-TOF LC/MS system (Agilent Technologies, USA). HPLC analysis was carried out on a C18 column (100 × 1.8 mm, i.d 2.1 *μ*m) at a flow rate of 0.4 mL/min. The mobile phase consisted of an aqueous solution and acetonitrile (7 : 3). The injection volume was 1 *μ*L, and the column temperature was set at 25°C. Mass analysis was performed by electrospray ionization (ESI) in the positive ion mode with the capillary voltage set at 3500 V. The gas temperature, sheath gas temperature, and sheath gas flow were set to 250°C, 350°C, and 11 L/min, respectively.

### 2.4. Atopic Dermatitis Preparation in BALB/c Mice

Eight-week-old BALB/c female mice were purchased from the Animal Facility in Guilin Medical University. All animal experiments were approved by the Animal Experimental Ethics Committee of Guilin Medical University (SYXK Gui 2013-0001). These guidelines were in accordance with the internationally documented principles for laboratory use and care. All mice were housed in specific pathogen-free conditions at controlling temperature 20-25°C, and the relative humidity was 45%-65%, and they were freely fed a normal diet and water and maintained in a 12/12 h light/dark cycle. Mice were randomly divided into four groups (*n* = 8 animals/group) as follows: (1) normal group (without any treatment), (2) vehicle group (oxazolone+normal saline), (3) hydrocortisone (HC) group (oxazolone+26 mg/kg body weight HC), and (4) PFS group (oxazolone+50 mg/kg body weight PFS). PFS and HC dissolved in normal saline were orally administered with daily doses for 34 consecutive days, respectively. The vehicle group received an equal volume of normal saline in the same route. Atopic-like dermatitis was performed as previously described with some modifications [32–35]. In brief, mice were anesthetized by isoflurane inhalation and sensitized by topically applying 100 *μ*L of 1.5% oxazolone solution (acetone : olive oil = 4 : 1) to shaved abdomen skin (2 cm × 2 cm); 2 weeks later, dermatitis was induced by the application of oxazolone on both sides of the ear with 15 *μ*L of 0.1% oxazolone solution every 48 h for 10 times (ten-time challenge). The ear thickness was measured with a dial thickness gauge (Peacock, Japan) from the day of sensitization until the end of this experiment. On day 20, the animals were anesthetized by isoflurane inhalation, and the inflamed ears were macroscopically scored by two independent laboratory staff as described elsewhere [36]. Then, the animals were euthanized, and their blood and ear samples were collected for further assessment.

### 2.5. Histological Analysis

Mouse skin tissues were fixed with 4% paraformaldehyde for 1 h, dehydrated with 30% sucrose solution overnight at 4°C, then embedded in OCT and stored under -20°C. Cryosections of 5 *μ*m thickness were fixed in cold acetone, then rehydrated in graded ethanol and PBS and used for hematoxylin-eosin (H&E) staining and toluidine blue staining. Mast cells were counted, and epidermal thickness was measured using ImageJ analysis software. For immunohistochemistry (IHC), 0.3% H_2_O_2_-methanol incubated sections were blocked with goat serum at 37°C for 30 min and incubated overnight with the primary antibodies or isotype controls at 4°C, after vigorously washed; a secondary antibody was added and incubated at 37°C for 30 min followed by diaminobenzidine and counterstained with hematoxylin. Specific markers were examined by light microscopy (Olympus, Tokyo, Japan) at 100x or 400x magnification.

### 2.6. BMDM Preparation

BMDMs were isolated and differentiated using standard protocols [37]. Bone marrow cells were obtained by flushing the tibia and femur taken from 6-8-week-old BALB/c mice; the cells were cultured in *α*-MEM containing 4.5 g/L of glucose, 10% FCS, penicillin (100 U/mL), and streptomycin (100 *μ*g/mL) for 24 h. Suspended cells were differentiated in *α*-MEM with 30 ng/mL M-CSF for 7 days to harvest BMDMs. The culture medium was changed every 3 days.

### 2.7. Cell Viability Assay

For cell viability determination, BMDMs were plated in 96-well plates at a density of 5 × 10^4^ cells/well with or without PFS for 24 h. Then, the cell medium was discarded and 100 *μ*L of 5 mg/mL MTT was added and incubated for 4 h. Then, the supernatants were removed, and the formazan formed in the intact cells was dissolved with 150 *μ*L/well dimethyl sulfoxide at 37°C for 10 min. The absorbance was measured by a spectrophotometer at 490 nm.

### 2.8. LPS-Induced M1 Macrophage Polarization

BMDMs were plated in 6-well plates at a density of 1 × 10^6^ cells/well. The cells were divided into (1) normal group; (2) vehicle group (100 ng/mL LPS); and (3) PFS group (100 ng/mL LPS+PFS). The PFS groups were pretreated with PFS at specified concentrations for 12 h, the normal and vehicle group received equal culture medium only, and then, the vehicle and PFS groups were stimulated with LPS (100 ng/mL) as indicated times. BMDMs were collected to determine the expression of NOS2, Arg1, and p-STAT3 using western blot.

### 2.9. Immunofluorescence Staining

BMDMs (1 × 10^5^ cells/well) were inoculated in a 12-well plate. When the cells grow to 70%-80%, immunofluorescence staining was performed as follows: after being rinsed 3 times with PBS, cells were fixed with 4% paraformaldehyde at room temperature for 10 min, and the cells were treated with 0.1% Triton X-100 for 10 minutes and blocked with 5% goat serum in a 37°C incubator for 1 h. These cells were incubated with p65 at 4°C overnight, then incubated with a fluorescent-labeled secondary antibody (1 : 50) for 30 min in a 37°C incubator. Cells were counterstained with DAPI for 1 min. The marker-positive cells were observed under a fluorescence microscope.

### 2.10. Western Blot Analysis

The ear tissues ground in liquid nitrogen or cells were extracted with RIPA buffer containing 50 mM Tris-HCl, 1% Triton X-100, 0.2% sodium deoxycholate, 0.2% SDS, 1 mM EDTA, 100 *μ*M PMSF, protease inhibitor cocktail, and phosphatase inhibitors. Protein concentration was determined by BCA assay. Equal amounts of proteins were subjected to 10% SDS-PAGE (Bio-Rad, USA) and subsequently transferred to polyvinylidene difluoride membranes (PVDF, Millipore, Germany) using a semidry electroblotting apparatus. The membranes were blocked with 5% BSA for 2 h at room temperature and incubated overnight at 4°C with primary antibodies against NOS2 (1 : 100), MCP-1 (1 : 200), STAT3 (1 : 100), Arg1 (1 : 100), CD163 (1 : 100), p-STAT3 (1 : 2000), and GAPDH (1 : 5000). After three washes in TBST, the membranes were incubated with corresponding HRP-conjugated secondary antibodies, and protein bands were visualized by ECL solution. The band density analysis was done using Image Lab.

### 2.11. Enzyme-Linked Immunosorbent Assay (ELISA)

Plasma samples were obtained by centrifuging at 3,000 rpm for 10 min and stored at -80°C until required for assay, and the ear tissues were grinded in liquid nitrogen. The tissue was lysed in RIPA buffer (Highly Efficient Solitaire, Solaibao, China) containing 100 *μ*M PMFS (Solaibao, China) and protease inhibitor cocktail (MCE, USA), and the supernatant was collected after centrifugation and stored at -80°C until analysis. The levels of IgE, IgG1, and IgG2a in plasma and TNF-*α*, MCP-1, IL-4, IL-6, IL-10, and IL-22 (Thermo Fisher) in ear tissues were determined according to the manual of ELISA kit.

### 2.12. Relative Gene Expression

Mouse ear tissues were pulverized in liquid nitrogen, then dissolved into Trizol solution immediately. Total RNA was extracted according to the manufacturer's protocol (Tiangen, China), and cDNA was synthesized using FastKing First-Strand Synthesis System (Tiangen, China). Serial amplification was carried out according to the 2X Taq PCR Master Mix RT reaction system. The band density was calculated using ImageJ analysis software and normalized to GAPDH.

### 2.13. Statistical Analysis

The statistical analysis of the data was carried out by software SPSS17.0 (International Business Machines Corporation, Armonk, New York, USA), and the graphs were made with software GraphPad Prism 5 (GraphPad Software Inc., La Jolla, CA, USA). The results were expressed as the means ± SD or the means ± SEM. Statistical significance was evaluated by the one-way analysis of variance (ANOVA) for comparison between multiple groups. *p* < 0.05 was considered statistically significant.

## 3. Results

### 3.1. Determination of Periplocin Content in PFS

**Figure 1:**
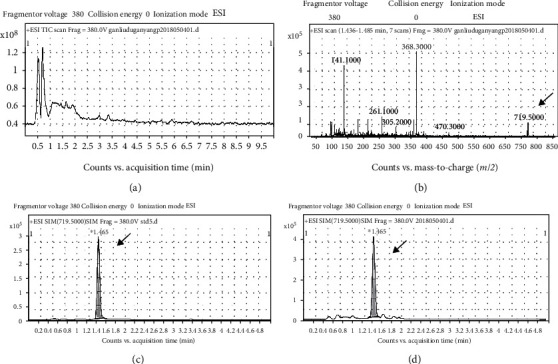
Determination of periplocin in PFS: (a) TIC of PFS; (b) TIC showing the peak of periplocin in PFS; (c) typical XIC showing the peak of periplocin standard; (d) typical XIC showing the peak of periplocin in PFS.

For identification and quantification of the active components in PFS, we performed LC-MS analysis of periplocin, one of active ingredients of PFS. Total ion chromatogram (TIC) on PFS was performed (Figure 1(a)), and the peak (719.5) of periplocin in PFS was identified in TIC within 1.436-1.485 min (Figure 1(b)). The peaks of periplocin in the standard and PFS are found at 1.465 min in the extracted ion chromatograms (XIC) (Figures 1(c) and 1(d)). The content of periplocin in PFS was 0.4% (g/g). Two unidentified peaks were found in front of the periplocin peak, which require further isolation and identification (Figure 1(a)).

### 3.2. PFS Treatment Alleviated AD-Like Symptoms

**Figure 2:**
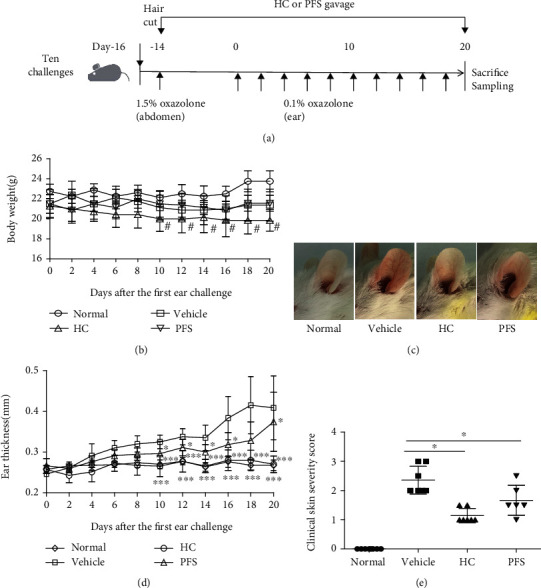
PFS improved the development of atopic dermatitis- (AD-) like symptoms in oxazolone-induced BALB/c mice (ten-time challenge). BALB/c female mice were sensitized as described in Materials and Methods. The PFS and HC groups were intragastrically administered with PFS or HC continuously for 34 days. (a) Experimental protocol; (b) clinical features of AD-like skin lesions at day 20; (c) thickness of the left ear of the mouse; (d) clinical score of AD-like skin lesions at day 20. Data are shown as mean ± SEM (*n* = 6-8), ^∗^*p* < 0.05, ^∗∗^*p* < 0.01, and ^∗∗∗^*p* < 0.001 versus the vehicle group; ^#^*p* < 0.05 versus the PFS group.

**Figure 3:**
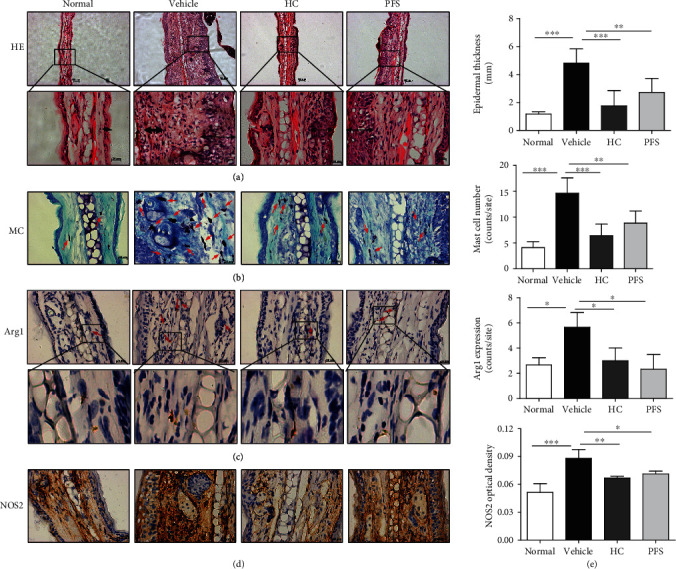
PFS improved the histology of atopic dermatitis (AD) in mice (ten-time challenge). Tissues were excised, fixed in 10% formaldehyde for 1 h, embedded in OTC, and sectioned. (a) HE staining (100x; 400x); arrow indicates epidermal exfoliation; (b) toluidine blue staining (400x); arrows: mast cells; (c) Arg1 (100x; 400x); arrows: Arg1-positive macrophages; (d) NOS2 (400x); (e) epidermal thickness, number of mast cells, Arg1-positive cells, and NOS2 optical density were detected. ^∗^*p* < 0.05 and ^∗∗^*p* < 0.01 versus the vehicle group.

Atopic dermatitis is characterized by a chronic, pruritic inflammatory skin disease; to induce a chronic AD model, mice were sensitized by applying 1.5% oxazolone to the abdomen. Mice were subsequently resensitized by applying 0.1% oxazolone to the ears every other day for ten times (ten-time challenge). The PFS-treated mice received a daily dose of 50 mg/kg body weight PFS via intragastric gavage, and mice receiving 26 mg/kg body weight HC were used as positive controls. A schematic experimental protocol is depicted as shown in Figure 2(a). Body weight was recorded every other day, and weight loss appeared in all the oxazolone-treated group. Mice given PFS showed no significant weight loss compared to vehicle-treated mice but experienced remarkably decreased body weight loss when compared to HC-treated mice (Figure 2(b)). Mice administered with PFS and HC showed significant lower levels of ear thickness compared to mice treated with vehicle (both *p* < 0.05) (Figures 2(c) and 2(d)). Typical AD symptoms were evaluated at the end of the experiment, including erythema, edema, epidermal exfoliation, scabs, and xerosis, and treatment with PFS significantly improved these symptoms (*p* < 0.05), as did HC treatment (Figure 2(e)). The pathological histological changes in AD mice were examined at the end of the experiment. The skin lesions are characterized by marked inflammatory cell infiltration, epidermal hyperplasia, and dermal edema. In particular, inflammatory infiltrates, specifically the number of mast cells in histological analysis, were increased (Figure 3(a)). However, treatment of the ear with PFS attenuated these inflammatory responses as did HC (Figures 3(a)–3(c)). Effects of PFS on the expression of M1 and M2 macrophage-specific markers were evaluated by IHC. Oxazolone challenge to the ear significantly increase the Arg1 and NOS2 expression compared with the normal group; PFS dramatically reduced the expression of Arg1 and NOS2 as did the HC treatment (data not shown).

### 3.3. PFS Abrogated Plasma IgE and Skin Inflammation in Oxazolone-Induced AD Mice

**Figure 4:**
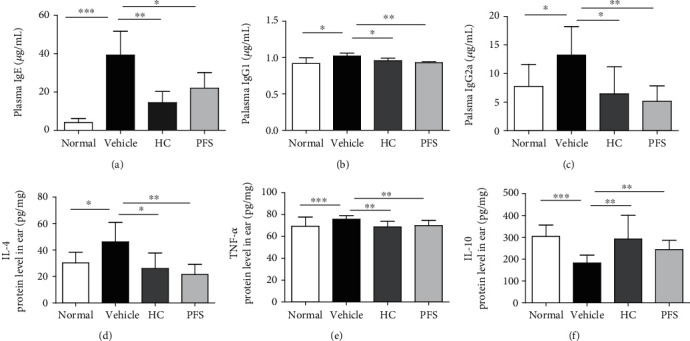
Effects of PFS on the production of IgE and skin inflammation in oxazolone-induced dermatitis (ten-time challenge). Data are expressed as mean ± SD (*n* = 6). ^∗^*p* < 0.05 and ^∗∗^*p* < 0.01 compared with the vehicle group.

To investigate the effects of PFS on allergy-related immunoglobulin, the plasma levels of Th2-mediated IgE and IgG1 and Th1-mediated IgG2a were measured in ten-time-challenged mice. In the oxazolone treatment group, the level of IgE, IgG1, and IgG2a was increased as compared with the vehicle group. However, oral administration of PFS alleviated the level of IgE, IgG1, and IgG2a as compared with the oxazolone treatment group ((Figures 4(a)–4(c))). The effects of PFS on the IgE, IgG1, and IgG2a levels were comparable to those of HC. These results indicate that PFS may inhibit the development of AD by lowering the level of IgE, IgG1, and IgG2a. The levels of Th1 and Th2 mediators were measured in lesional skin. PFS alleviated the levels of IL-4 and TNF*α*, while it augmented the levels of IL-10 (Figures 4(d)–4(f)).

Since the amount of hapten was implicated in the response of AD, we also performed oxazolone-induced dermatitis by five-time challenges with a higher concentration at 0.5% oxazolone solution. We sought to evaluate the protective effect of PFS on the general characteristics of AD, including high levels of plasma IgE, Th1/2, or M1/M2-related factors. Five-time-challenged mouse skin with higher concentration of oxazolone exhibited sever erythema and edema. However, consistent with the ten-time challenge model, PFS effectively suppressed AD-related skin swelling and erythema compared with vehicle mice, as did HC (Supplementary Figures S(a) and (b)). As shown in Supplementary Figures S(c–g), ELISA showed that oxazolone challenge significantly increased the plasma IgE levels in mice; meanwhile, the expression of TNF-*α*, IL-4, MCP-1, and IL-6 in mouse ear tissue also manifested a remarkable upward trend compared to matched controls. However, PFS treatment could noticeably restore this increment, as did HC. In addition, IL-22 was significantly increased in the same manner as Th1 and Th2 cell cytokines but significantly decreased by PFS administration, as did HC ((Supplementary Figure S(h)).

### 3.4. PFS Downregulated the Protein Expression of Macrophage-Related Markers in AD Mice

**Figure 5:**
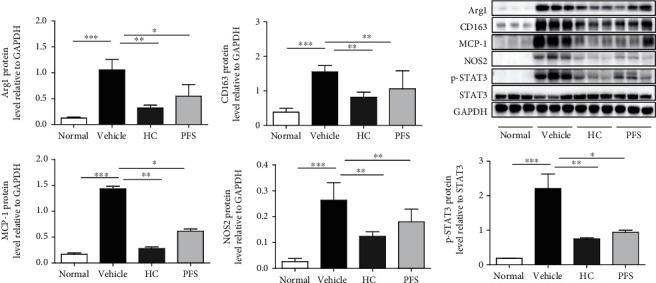
PFS suppressed the protein expression of macrophage factors in oxazolone-induced BALB/c mice (ten-time challenge). The expression of macrophage marker and STAT3 in AD skin lesion was evaluated by a western blot analysis. The histogram bars represent three independent experiments. Data are expressed as mean ± SD, ^∗^*p* < 0.05, ^∗∗^*p* < 0.01, and ^∗∗∗^*p* < 0.001 versus the vehicle group.

In order to evaluate the inflammatory mediator production in ten-time-challenged AD mice, we examined the protein level in the lesional ear using western blot. Oxazolone treatment induced a dramatic increase in the expression of M1 marker MCP-1 and NOS2 and M2 marker Arg1 and CD163 compared with the normal group (Figure 5). PFS significantly inhibited the expression of NOS2 (*p* < 0.01), MCP-1, Arg1 (*p* < 0.05), CD163 (*p* < 0.001), and p-STAT3 (*p* < 0.05) as did HC. These results suggested that PFS could downregulate the expression of both M1 and M2 markers that are associated with AD development. Interestingly, the similar inhibitory effects were also found in the HC group.

### 3.5. PFS Abrogated AD-Related M1/2 Macrophage-Related Gene Markers in AD Mice

**Figure 6:**
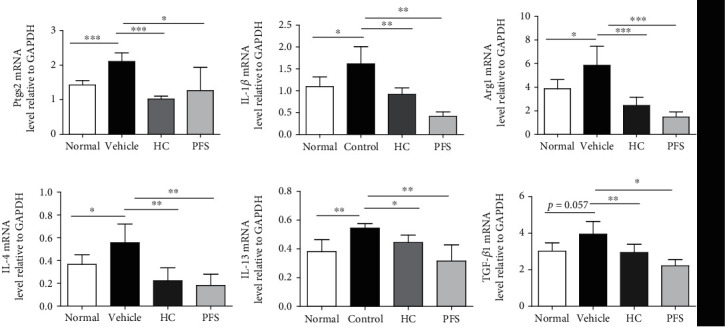
PFS inhibited mRNA expression of M1/2 macrophage in oxazolone-induced BALB/c mice (ten-time challenge). Expression of mRNA levels was determined by RT-PCR. The grey values were normalized to GAPDH and expressed as mean ± SD (*n* = 6), ^∗^*p* < 0.05, ^∗∗^*p* < 0.01, ^∗∗∗^*p* < 0.001 versus the vehicle group.

To assess the effects of PFS on changes in T cell and macrophage infiltrations in the ten-time challenge model, mRNA levels in ear tissues of M1 factors IL-1*β* and prostaglandin endoperoxide synthase2 (Ptgs2, coding COX2); M2 factors IL-4, IL-13, transforming growth factor- (TGF-) *β*1, and Arg1 were analyzed by RT-PCR (Figure 6). Oxazolone-challenged ears demonstrated dramatic increases in Ptgs2, IL-1*β*, Arg1, IL-4, IL-13, and TGF-*β*1 compared with the normal group. However, PFS treatment led to significantly lower mRNA levels of IL-1*β* (*p* < 0.05), IL-4 (*p* < 0.05), IL-13 (*p* < 0.01), and TGF-*β*1 (*p* < 0.05). Moreover, PFS significantly inhibited the expression of M1 macrophage-specific marker Ptgs2 (*p* < 0.05) and M2 macrophage-specific marker Arg1 (*p* < 0.05). Taken together, PFS inhibited M1/M2 mRNA expression in AD mice. Interestingly, the potential inhibitory effects were also found in the HC group.

### 3.6. PFS Suppressed LPS-Induced M1 Macrophage Polarization

**Figure 7:**
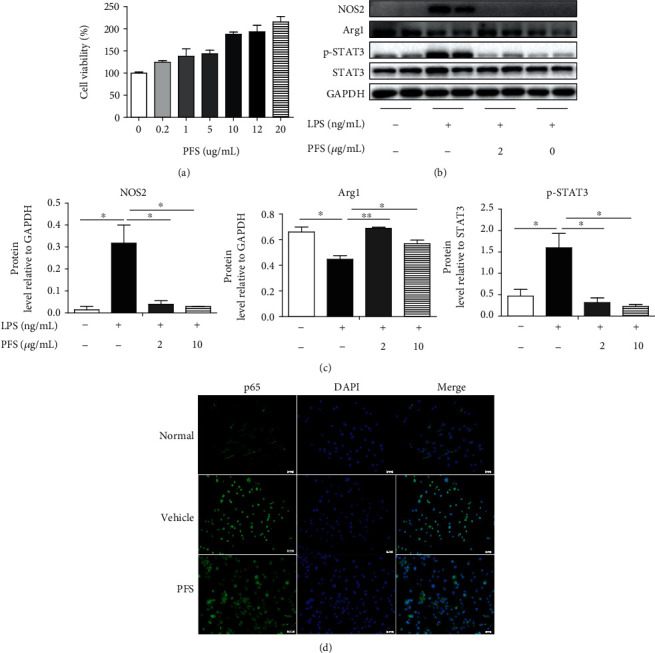
Immunomodulatory effects of PFS on LPS-induced M1 macrophage polarization. (a) BMDMs in 96-well plates (5 × 10^4^ cells/well) were cultured in the presence or absence of different concentrations of PFS for 24 h; then, 100 *μ*L of 5 mg/mL MTT was added and incubated for 4 h, the formazan formed in the intact cells was dissolved with 150 *μ*L/well dimethyl sulfoxide (DMSO) at 37°C for 10 min, the OD was measured at 490 nm, and cell viability was calculated (*n* = 6). (b) SDS-PAGE protein bands and (c) the optical densities; BMDMs in 6-well plates (1 × 10^6^ cells/well) were pretreated with PFS at 2 *μ*g/mL and 10 *μ*g/mL for 12 h and stimulated with LPS (500 ng/mL) for 0.5 h; then, protein samples were collected. The expression of macrophage-associated factors was detected using western blot (*n* = 3). The results were means ± SD, ^∗^*p* < 0.05 and ^∗∗^*p* < 0.01 versus the vehicle group. (d) Macrophage polarization and nuclear localization of p65 were performed by immunofluorescence staining. Scale bar, 20 *μ*m.

The effects of different concentrations of PFS on BMDMs viability were determined by the MTT method. As shown in Figure 7(a), we found that PFS promoted M-CSF-induced bone marrow-derived macrophage (BMDM) viability in a concentration-dependent manner. Next, we investigated the anti-inflammatory effects of PFS by evaluating macrophage polarization in BMDM with or without LPS stimulation. BMDMs were stimulated with LPS (100 ng/mL) for 12 h in the presence or absence of PFS at 2 and 10 *μ*g/mL. Protein expression levels during macrophage polarization were measured by western blot. PFS inhibited the expression of NOS2 (*p* < 0.05) and the phosphorylation of STAT3 (*p* < 0.05) and upregulated the Arg1 level in a dose-dependent manner (Figures 7(b) and 7(c)). The above results indicated that PFS treatment promoted macrophage proliferation and increased the expression of M2 factors but inhibited the expression of M1 factors, resulting in the suppression of LPS-induced M1 macrophage polarization. Hence, the inhibitory effect of PFS- on the LPS-induced BMDMs was deemed not to be attributable to its cytotoxicity. Because intracellular signaling proteins like STATs and NF-*κ*B, which are downstream of TLR4 signaling, regulate macrophage polarization, we next investigated whether PFS had any impact on NF-*κ*B nuclear translocation in BMDMs. As shown in Figure 7(d), in unstimulated BMDMs, p65 was localized mostly in the cytosol, and after LPS induction, p65 was almost completely concentrated in the nucleus. This activation-dependent translocation was suppressed to some levels by incubating BMDMs with PFS. These findings uncovered that PFS ameliorated LPS-induced M1 macrophage polarization by blockage of p65 translocation into the nucleus.

## 4. Discussion

Murine models are useful for understanding the pathogenic mechanisms and therapeutic drug discovery of AD. Dermatitis models induced by repeated application of protein haptens such as ovalbumin and mite antigens or chemical haptens such as trinitrochlorobenzene (TNCB), 2,4-dinitrofuorobenzene (DNFB), and 2,4-dinitrochlorobenzene (DNCB) on the skin of mouse or rat dorsal ear are the most commonly used AD models [32, 38–40]. In this study, PFS could significantly attenuate the occurrence of oxazolone-induced AD-like lesion by repeated application of oxazolone on the ears of BALB/c mice. PFS reduced the ear swelling and clinical symptoms in AD mice, inhibited the epidermal thickness and the infiltration of mast cells, and reduced IgE, IgG1, and IgG2a levels in plasma, Th1/2, and M1/2 factors in the ear tissue.

In this study, gene expression of TNF-*α*, IL-1*β*, IL-4 IL-13, and TGF-*β*1 increased in the ten-time challenge model, suggesting that repeated oxazolone application induced AD in a mixed Th1/Th2 pattern [41–43], and PFS significantly suppressed the protein expression of TNF-*α*, MCP1, IL-4, and IL-6 but augmented IL-10 protein levels. IL-22 played an important role in both acute and chronic AD lesions and positively associated with AD severity and participate in the increase of epidermal hyperplasia, dysfunction of barrier, and inhibition of epidermal differentiation [44–46]. PFS also suppressed the level of IL-22, suggesting its potential effects on the epidermal barrier function, which needs further study.

AD patients exhibit a mixed Th1/Th2 phenotype, and it is not surprising that mixed phenotype macrophages (M1/M2) were observed in AD [13]. Macrophages are known to accumulate rapidly and chronically in AD inflamed skin [12, 47]. M1 macrophages predominate during the early stage of inflammation, which mediate pathogen clearance and recruitment of other effector cells, while M2 macrophages prevail toward the end of inflammation [10]. In this study, the activated macrophages had a M1/M2 heterogeneous phenotype in the ear lesions, and M2 macrophage markers CD163 and Arg1 were highly expressed in the protein level, especially Arg1 which was found to be increased in the protein, gene, and histology levels. These results are also in agreement with the findings that Arg1 is a negative regulator in allergic contact dermatitis (ACD) [48, 49], and CD163 was present in the cells or sera of patients with psoriasis and AD [50, 51]. However, PFS downregulated the expression of macrophage factors CD163 and Arg1, suggesting its regulatory effect on the M2 macrophage factor in AD mice. Further studies could provide novel insights into the functional roles of CD163 and Arg1 during oxazolone-induced dermatitis. We assessed M1 factors NOS2 and Ptgs2, which are considered universal markers for evaluating the anti-inflammatory effect of pharmacological agents [52]. PFS reduced the protein expression of NOS2 in AD mice and inhibited Ptgs2 mRNA in AD mice. PFS treatment also downregulated the expression of MCP-1, which plays a pivotal role in mediating the attraction of leucocytes to sites of inflammation [53]. Activated STAT3 protein expression is found to be highly expressed in the skin of AD patients [54], and STAT3 is also activated by M2-polarizing signals (IL-4, IL-10, and IL-13) [55, 56]. However, STAT3 directly regulate oxidants and inflammatory mediators such as iNOS and COX-2 in the development of inflammation [57]. STAT3 activation can regulate a large number of downstream genes related to cell proliferation and immune diseases [58]. In this study, PFS reduced the activation of STAT3 in the ear tissue in both AD mice in vivo and macrophage polarization in vitro, showing the therapeutic and immunomodulatory effects of PFS on AD mice.

Interestingly, we observed that LPS exposure induced increased M1 polarity switch of macrophage marker NOS2 and lower expression of M2 macrophage marker ARG1 in BMDMs, which is consistent with the previous report [59], and we might find that the macrophage polarization response induced by LPS in vitro is only partially the same as the macrophage response in vivo; however, PFS and HC treatment attenuated the response of BMDMs to LPS stimuli.

It is generally accepted that proinflammatory stimulation could activate the NF-*κ*B signaling pathway [60]. In the course of inflammatory disease, NF-*κ*B activation controls the production of proinflammatory mediators, such as NO and its mediator protein iNOS, PGE2 and its mediator protein COX2, and cytokines IL-6 and TNF-*α* [61]. In the previous study, PFS was found to have inhibited the phosphorylation of p65 and its inhibitory protein (I*κ*B) [31]. We deservedly further investigate the inhibitory effects of PFS on this critical transcription factor. Firstly, we showed that PFS significantly enhanced the inhibitory effects on the release of NOS2 and MCP-1 and nuclear translocation of p65 in LPS-activated BMDMs, indicating that the anti-inflammatory effects of PFS are related to the NF-*κ*B signaling pathway.

## 5. Conclusions

In summary, PFS treatment could inhibit inflammatory response in AD-like dermatitis in BALB/c mice. PFS inhibited macrophage activation via downregulating mixed M1/2 macrophage factors and related transcription factors in AD mice. Moreover, PFS promoted M-CSF-induced bone marrow-derived macrophage viability, inhibited M1 macrophage factors, and regulated M2 macrophage factors, thus affecting the macrophage polarization in vitro. However, the specific mechanism of PFS on AD and its role in skin barrier-related factors such as filaggrin, the regulation of macrophage polarization, and its related pathways require further studies.

## Data Availability

The datasets used and/or analyzed in the current study are available from the corresponding author on reasonable request.

## Supplementary Materials

Supplementary Materials Supplementary Table: nucleotide sequences of primers used for polymerase chain reaction (RT-PCR) of mouse genes. Supplementary Figure: PFS suppressed plasma IgE and inflammatory factors in ear tissue in oxazolone-challenged BALB/c mice (five-time oxazolone challenge). Mice were sensitized by topically applying 100 *μ*L of 1.5% oxazolone solution (acetone : olive oil = 4 : 1) to shaved abdomen skin (2 cm × 2 cm); 2 weeks later, dermatitis was induced by application of oxazolone on both sides of the ear with 15 *μ*L of 0.5% oxazolone solution once every 72 h for 5 times. The PFS and HC groups were intragastrically administered with PFS or HC continuously for 29 days. (a) The thickness of mouse ear; (b) clinical features of AD-like skin lesions at day 15; (c) plasma IgE levels; (d–h) levels of inflammatory factors in ear tissue proteins. Results are expressed as mean ± SD (*n* = 6-8), ^∗^*p* < 0.05, ^∗∗^*p* < 0.01, and ^∗∗∗^*p* < 0.001 compared with the vehicle group.
