# Supplementary figures and images for: Saponin from Periploca forrestii Schltr Mitigates Oxazolone-Induced Atopic Dermatitis via Modulating Macrophage Activation
Source: Mediators Inflamm. 2020 Oct 15;2020:4346367. doi: 10.1155/2020/4346367 (PMC7584956; doi:10.1155/2020/4346367)

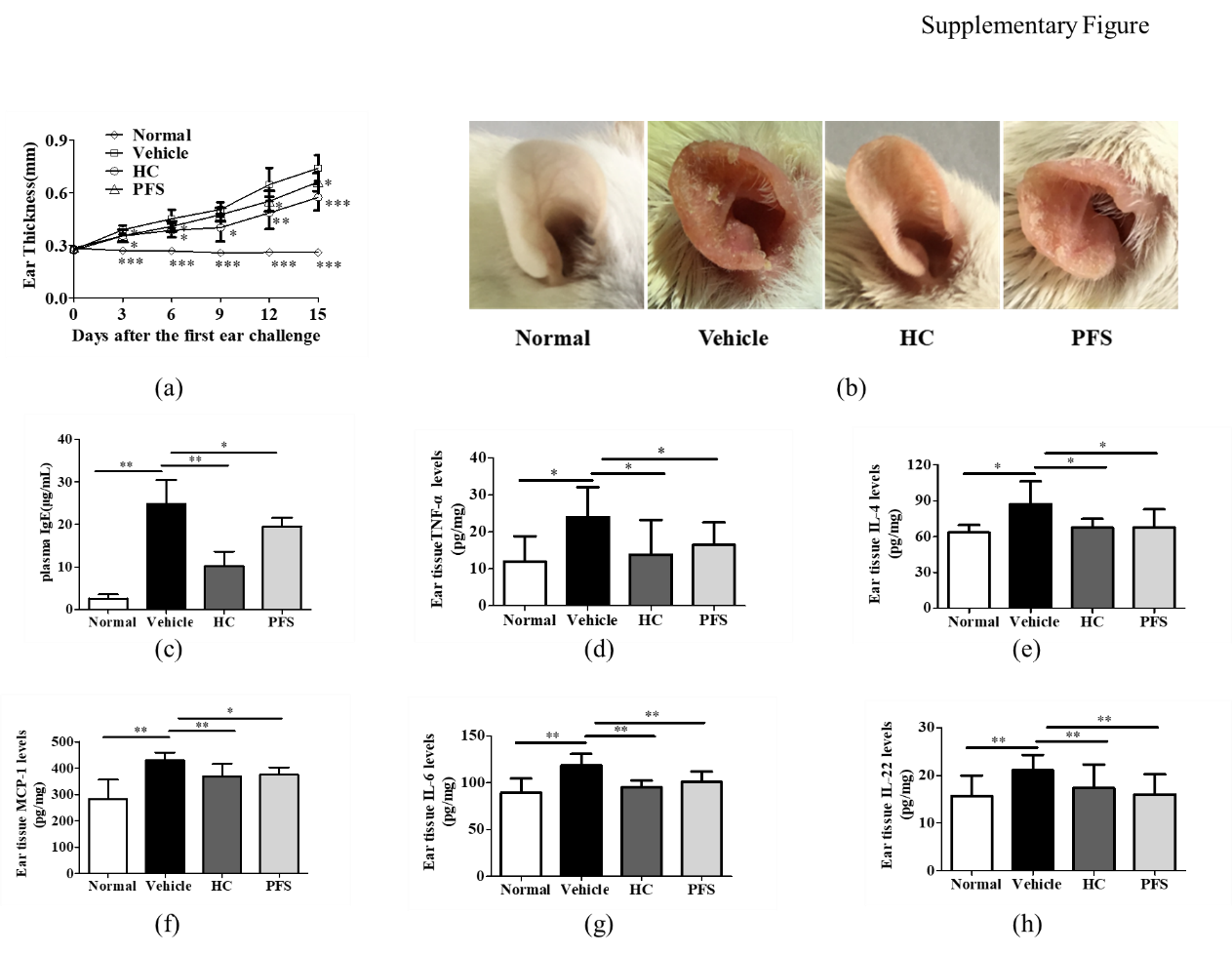

Supplement: Supplementary Materials — Supplementary Table: nucleotide sequences of primers used for polymerase chain reaction (RT-PCR) of mouse genes. Supplementary Figure: PFS suppressed plasma IgE and inflammatory factors in ear tissue in oxazolone-challenged BALB/c mice (five-time oxazolone challenge). Mice were sensitized by topically applying 100 μL of 1.5% oxazolone solution (acetone : olive oil = 4 : 1) to shaved abdomen skin (2 cm × 2 cm); 2 weeks later, dermatitis was induced by application of oxazolone on both sides of the ear with 15 μL of 0.5% oxazolone solution once every 72 h for 5 times. The PFS and HC groups were intragastrically administered with PFS or HC continuously for 29 days. (a) The thickness of mouse ear; (b) clinical features of AD-like skin lesions at day 15; (c) plasma IgE levels; (d–h) levels of inflammatory factors in ear tissue proteins. Results are expressed as mean ± SD (n = 6-8), ∗p < 0.05, ∗∗p < 0.01, and ∗∗∗p < 0.001 compared with the vehicle group. [file 4346367.f1.docx]
